# Metabolomics analysis of plasma samples of patients with fibromyalgia and electromagnetic sensitivity using GC–MS technique

**DOI:** 10.1038/s41598-022-25588-2

**Published:** 2022-12-19

**Authors:** Cristina Piras, Monica Pibiri, Stella Conte, Gabriella Ferranti, Vera Piera Leoni, Sonia Liggi, Martina Spada, Sandro Muntoni, Pierluigi Caboni, Luigi Atzori

**Affiliations:** 1 Department of Biomedical Sciences, Clinical Metabolomics Unit, University of Cagliari, Blocco A, Cittadella Universitaria, Monserrato, CA Italy; 2 Department of Education, Psychology and Philosophy, University of Cagliari, Cagliari, Italy; 3 Department of Metabolism, Digestion and Reproduction, Imperial College London, London, UK; 4 Department of Life and Environmental Sciences, University of Cagliari, Cagliari, Italy

**Keywords:** Biomarkers, Diseases

## Abstract

Fibromyalgia (FM) is a chronic and systemic condition that causes widespread chronic pain, asthenia, and muscle stiffness, as well as in some cases depression, anxiety, and disorders of the autonomic system. The exact causes that lead to the development of FM are still unknown today. In a percentage of individuals, the symptoms of FM are often triggered and/or exacerbated by proximity to electrical and electromagnetic devices. Plasma metabolomic profile of 54 patients with fibromyalgia and self-reported electromagnetic sensitivity (IEI-EMF) were compared to 23 healthy subjects using gas chromatography-mass spectrometry (GC–MS) coupled with multivariate statistical analysis techniques. Before the GC–MS analysis the plasma samples were extracted with a modified Folch method and then derivatized with methoxamine hydrochloride in pyridine solution and N-trimethylsilyltrifuoroacetamide. The combined analysis allowed to identify a metabolomic profile able of distinguishing IEI-EMF patients and healthy subjects. IEI-EMF patients were therefore characterized by the alteration of 19 metabolites involved in different metabolic pathways such as energy metabolism, muscle, and pathways related to oxidative stress defense and chronic pain. The results obtained in this study complete the metabolomic "picture" previously investigated on the same cohort of IEI-EMF patients with ^1^H-NMR spectroscopy, placing a further piece for better understanding the pathophysiological mechanisms in patients with IEI-EMF.

## Introduction

Fibromyalgia (FM) is a condition characterized by a constellation of symptoms, including chronic pain, depression, anxiety, autonomic disturbance, fatigue, and memory and sleep dysfunction^1^. These numerous symptoms of functional and emotional origin seriously compromise the life quality of patients making the treatment harder and difficult^2^. At present, FM patients are diagnosed using the 2016 revised FM criteria^3^ based on the Fibromyalgia Research guideline^4^. According to these revised diagnostic criteria, fibromyalgia may be diagnosed in adults based on: (1) presence of generalized pain, defined as pain in at least 4 of 5 regions, (2) presence of symptoms of similar intensity level for at least 3 months, (3) widespread pain index (WPI) ≥ 7 and symptom severity scale (SSS) score ≥ 5 or WPI of 4–6 and SSS score ≥ 9; in addition, (4) a diagnosis of fibromyalgia does not exclude the presence of other clinical important illness^3^. However, the key causalfactors/mechanisms involved in FM development have not been identified yet^5^ and the lack of objective parameters to diagnose the pathology renders the discovery of more effective and safer diagnostic biomarkers urgently needed. Sometimes, FM is associated to electrosensitivity (EHS)^6^. EHS is described as a multi-organ adverse reaction to electromagnetic field (EMF), characterized by a wide range of unspecific symptoms. They can vary with intensity and duration and are experienced as a result of exposure in the workplace or home to EMF emitted by various sources, whether low or high frequency. Since the 60s, in countries of East Europe, there were reports of a new workplace disease defined as “microwave sickness”^7^: these cases involved thousands of workers in the manufacture, inspection, repair, and maintenance of microwave equipment such as radars and radio/TV stations. These reports have been extended to mobile phones in the last forty years. Researchers generally outline three characteristic syndromes: 1) neurological and/or asthenic: heaviness of head, fatigue, irritability, sleepiness, memory loss, and electroencephalography changes; 2) autonomic vascular changes: sweating, dermographism, blood pressure changes; 3) cardiac: heart pains and electrocardiography changes. Notably, workers exposed for periods above five years exhibited greater symptomatology. In addition, ceasing work was found to bring about a stabilization or improvement of symptoms^7^. The prevalence of EHS may continue to rise in the future, coinciding with the increasing exposure to local and global wireless networks. This could lead to an increased interest in the scientific community for the discovery of specific biomarkers. To better understand the pathogenesis of FM and EHS and to identify disease-specific biomarkers, we recently have compared the metabolomic profile between patients with FM and Idiopathic environmental intolerance attributed to electromagnetic field (IEI-EMF)^8^ and healthy subject (controls) by using ^1^H-NMR spectroscopy and multivariate statistical analysis. Self-reported IEI-EMF patients are subjects characterized by the amplification of fibromyalgia symptomatology in association with the proximity of EMF source exposure^9^.

Data obtained have shown a different plasma metabolomics profile between IEI-EMF and control subjects with the first being characterized by higher levels of metabolites mainly involved in oxidative stress defense, pain development and muscle metabolism. To better define and characterize the physiopathological mechanisms associated to the IEI-EMF, in the present study we have compared the plasma metabolomics profile of same IEI-EMF patients and control subjects studied by Piras et al.^8^ by using the mass spectrometry gas chromatography (GC–MS) analysis. GC–MS is a highly sensitivity technique that allows detection of many metabolites in complex biological samples. GC–MS and ^1^H-NMR provide complementary information about different metabolites, so that the integration of both techniques can be a major advantage to obtain a more holistic view of the metabolome^10^.

## Results

### Psychosocial descriptors

Patients had to fill in a questionnaire and the psychological tests according to the European EMF 2016 Guideline^11^.

In addition to the tests previously administered to IEI-EMF patients^8^, the PAI test allowed to evaluate further clinical and psychological characteristics such as: anxiety, depression, related anxiety disorders (like obsessive–compulsive disorders (OCD), phobias, etc.) and emotional instability. No differences between groups for clinical variables (e.g., depression, anxiety, anxiety correlates and emotional instability) were observed (p ≤ 0.05) and all subjects were located under the clinical cut-off (Supplementary Table S1 a) and b)).

### Metabolomics: multivariate statistical analysis

Principal Component Analysis (PCA) was then carried out to visualize the global distribution of samples and to highlight possible outliers. None of the samples had to be removed as outliers. Additionally, the quality control (QC), featuring a mix of all samples, was positioned in the middle of the PCA scatter plot indicating a reliable performance and reproducible of the GC–MS analysis (Figure S1).

**Figure 1:**
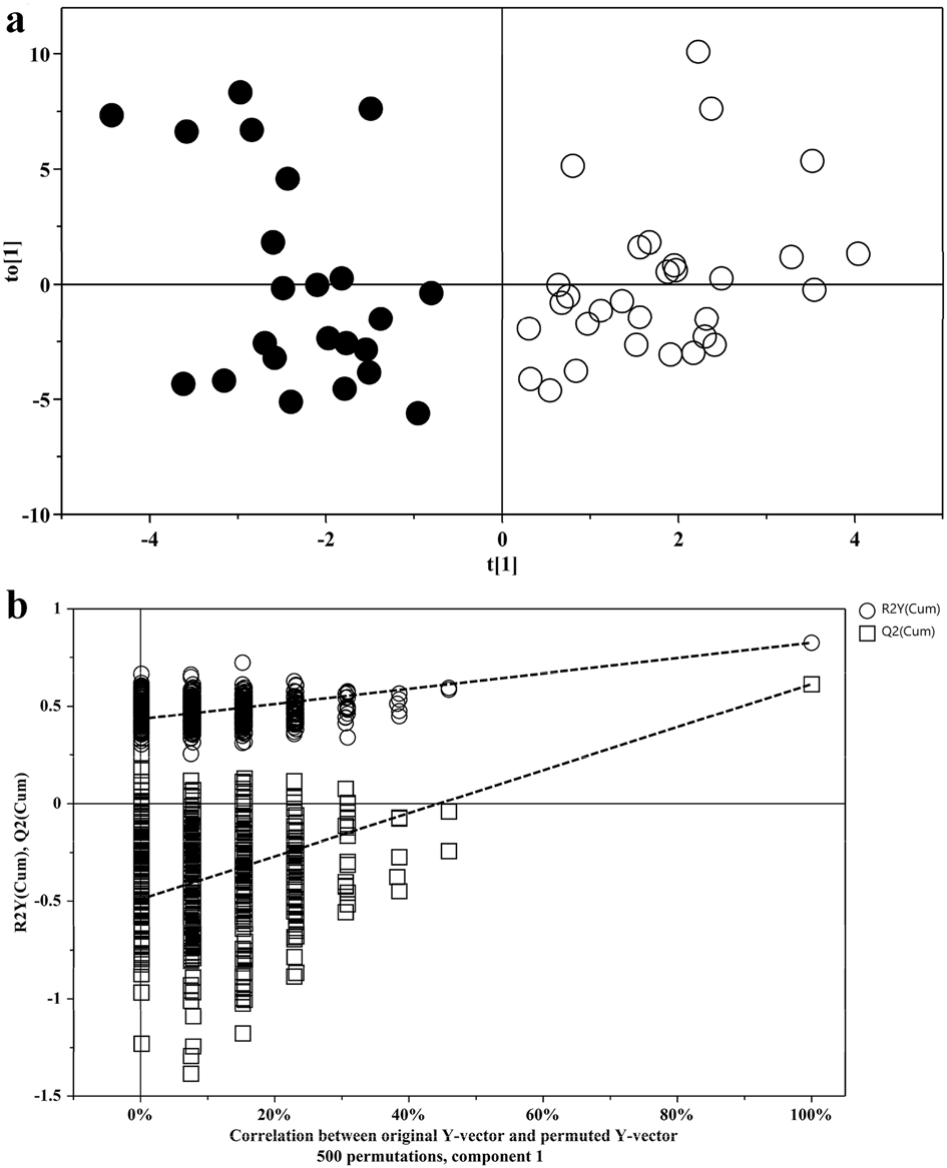
**(a)** OPLS-DA scores plot: controls (full circle), IEI-EMF subjects (open circle). (**b)** Validation plots of OPLS-DA model using a permutation test (n = 500). The horizontal axis shows the correlation between the permuted and actual data, while the vertical axis displays the cumulative values of R2 and Q2. The intercept gives an estimate of the overfitting phenomenon.

A supervised OPLS-DA analysis was subsequently conducted on the same dataset. OPLS-DA scores plot showed a clear separation based on the metabolomics profile between IEI-EMF subjects and controls (Fig. 1a). The optimum OPLS-DA model was established with two predictive components and one orthogonal component, with R2X(cum) of 0.456, R2Y(cum) of 0.825 and Q2 of 0.614. The validity of the OPLS-DA model was evaluated through a permutation test (Fig. 1b) using 500 cross validations.

**Figure 2:**
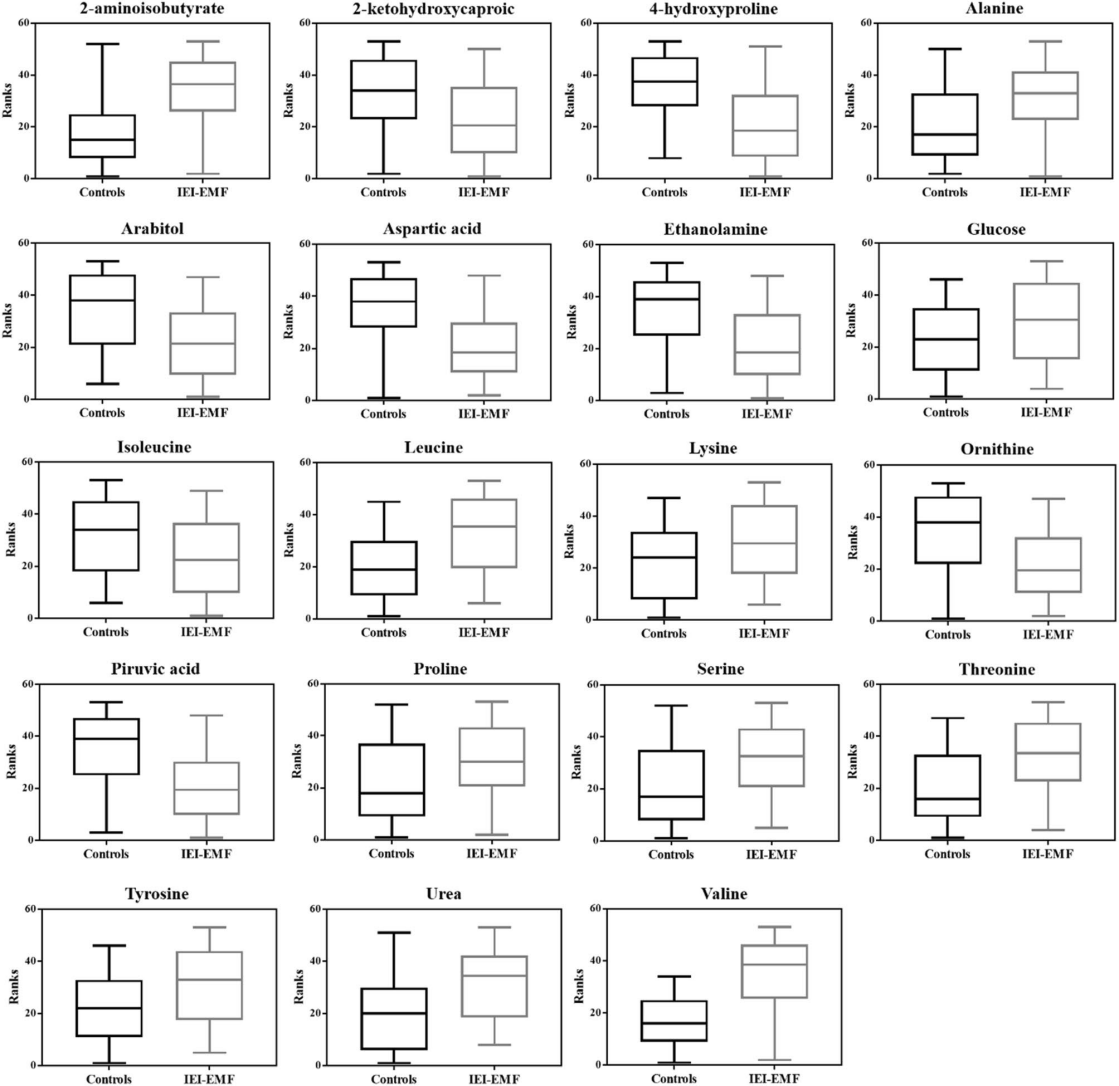
Statistically significant metabolites in IEI-EMF subjects compared to controls. The resulted metabolites are shown and expressed in the graphs y-axis as ranks (data transformation in which numerical or ordinal values are replaced by their rank when the data are sorted).

The metabolites characterized by VIP > 1 (Variable Influence on Projection) potentially responsible for the separation of IEI-EMF subjects from controls was evaluated with a Mann–Whitney U test with a Benjamini–Hochberg correction. Metabolites with a VIP > 1 significantly altered in the two groups are shown in Table 1 and their relative concentrations (Table S2) compared using box and whisker plots. (Fig. 2). As shown in Fig. 2, IEI-EMF subjects were characterized by a higher level of 2-aminoisobutyrate, alanine, glucose, leucine, lysine, proline, serine, threonine, tyrosine, urea, valine, and lower levels of 2-ketohydroxycaproic, 4-hydroxyproline, arabitol, aspartic acid, ethanolamine, isoleucine, ornithine, and pyruvic acid compared to controls. The receiver operating characteristic (ROC) plot was built by combining the discriminant metabolites (Figure S2). The area under the curve (AUC) was 0.876 (95% CI: 0.767–0.979), indicating the good predictive accuracy of the model. The predicted class probabilities (average of the cross-validation) for each sample using the 19 metabolites model are shown in Figure S3. The average accuracy based on 100 cross validations was 0.827 (Figure S4).

**Table 1:** Endogenous metabolites from plasma samples detected by GC-MS.

Metabolites	RT(min)	Extracted Ion (m/z)	VIP values	*p*-value	*p*-value correct*
Lactic acid	8.88	117	0.57	0.0770	-
**Pyruvic acid**	**9.03**	**217**	**1.44**	**0.0003**	**0.0087**
Glycolic acid	9.36	173	0.92	0.1675	-
**Alanine**	**9.74**	**116**	**1.26**	**0.0087**	**0.0326**
α-Hydroxybutyric acid	10.16	131	0.42	0.8379	-
m-Cresol	10.63	165	0.63	0.0740	-
β-Hydroxybutyric acid	10.68	117	0.61	0.0866	-
**2-Aminoisobutyrate**	**10.82**	**130**	**1.53**	** < 0.0001**	**0.0022**
**Proline**	**11.00**	**70**	**1.03**	**0.0314**	**0.0457**
**Valine**	**11.47**	**144**	**1.47**	** < 0.0001**	**0.0043**
**2-Ketohydroxycaproic**	**11.52**	**200**	**1.19**	**0.0043**	**0.0283**
**Urea**	**11.64**	**189**	**1.08**	**0.0067**	**0.0304**
**Ethanolamine**	**11.72**	**174**	**1.41**	**0.0007**	**0.0152**
**Serine**	**12.15**	**116**	**1.29**	**0.0111**	**0.0348**
**Leucine**	**12.28**	**158**	**1.37**	**0.0007**	**0.0174**
Glycerol	12.30	205	0.74	0.4900	-
**Isoleucine**	**12.59**	**158**	**1.16**	**0.0156**	**0.0370**
**Threonine**	**12.66**	**117**	**1.39**	**0.0010**	**0.0217**
Glycine	12.80	174	0.40	0.1656	-
Succinic acid	12.97	247	1.05	0.2494	-
Glyceric acid	13.10	189	0.60	0.3049	-
**Aspartic acid**	**14.99**	**232**	**1.14**	**0.0004**	**0.0130**
**4-Hydroxyproline**	**15.10**	**158**	**1.34**	** < 0.0001**	**0.0065**
Malic acid	15.21	233	0.36	0.8658	-
Threitol	15.35	217	0.14	0.0579	-
Pyroglutamic acid	15.72	156	0.74	0.0632	-
Threonic acid	15.81	292	0.69	0.0595	-
Creatinine	16.06	115	0.66	0.5871	-
Phenylalanine	16.12	120	0.37	0.7555	-
**Ornithine**	**16.45**	**142**	**1.22**	**0.0033**	**0.0261**
Glutamine	16.78	246	0.78	0.9362	-
Lauric acid	17.27	257	1.50	0.1169	-
**Arabitol**	**17.80**	**217**	**1.51**	**0.0026**	**0.0239**
Citric acid	18.97	273	0.85	0.2077	-
1,5-Anhydroglucitol	19.33	217	0.64	0.6371	-
Fructose	19.33	103	1.01	0.1302	-
Mannose	19.59	319	0.66	0.4462	-
**Glucose**	**19.71**	**205**	**1.07**	**0.0046**	**0.0500**
**Tyrosine**	**19.85**	**179**	**1.15**	**0.0252**	**0.0413**
**Lysine**	**20.01**	**174**	**1.03**	**0.0255**	**0.0435**
Inositol	20.28	318	0.40	0.1047	-
Maltotriose	20.38	217	0.69	0.4072	-
Palmitic acid	21.38	313	0.76	0.5390	-
Myo-inositol	21.60	305	0.40	0.6888	-
Uric acid	21.72	441	1.01	0.1087	-
Tryptophan	22.91	202	0.62	0.6758	-

– not measured; *with Benjamini–Hochberg correction.

Significant values are in [bold].

**Figure 3:**
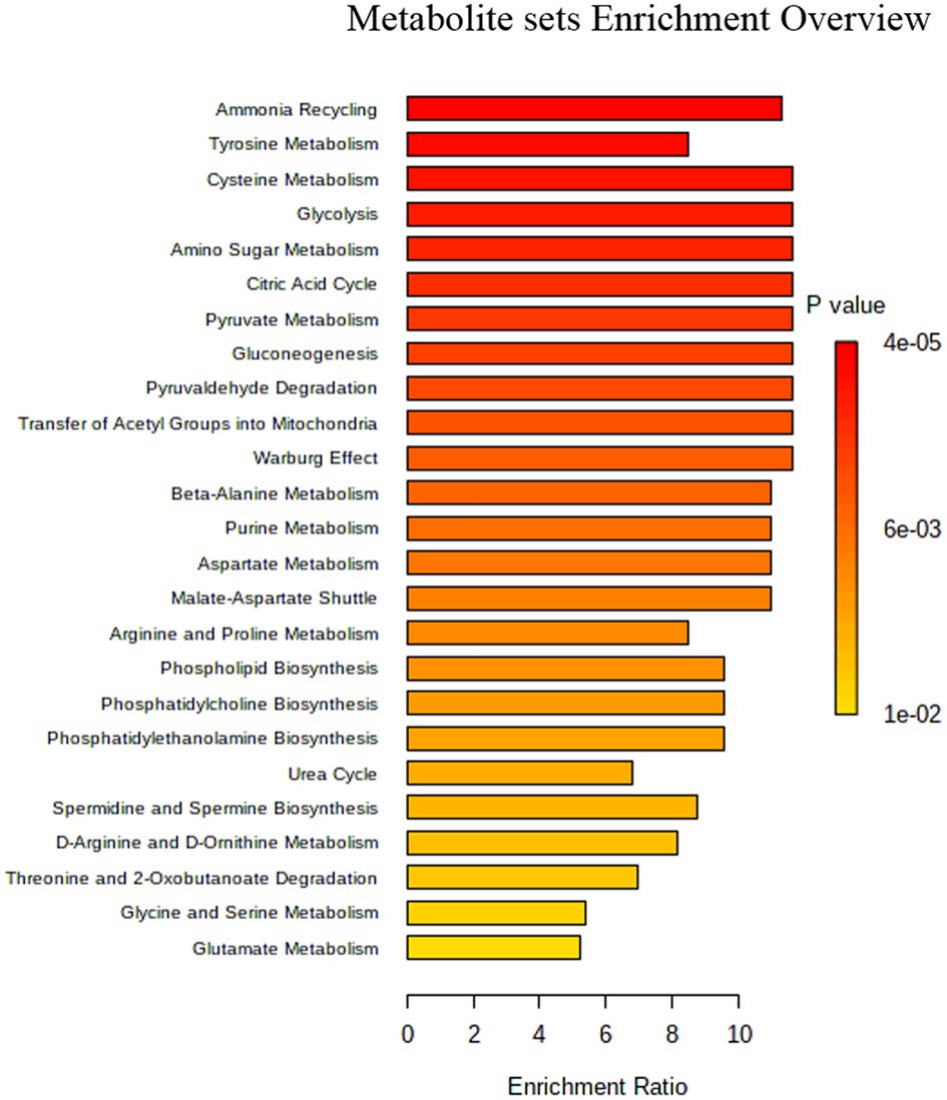
Enrichment analysis. List of the most significantly discriminant pathways between IEI-EMF subjects and controls.

**Figure 4:**
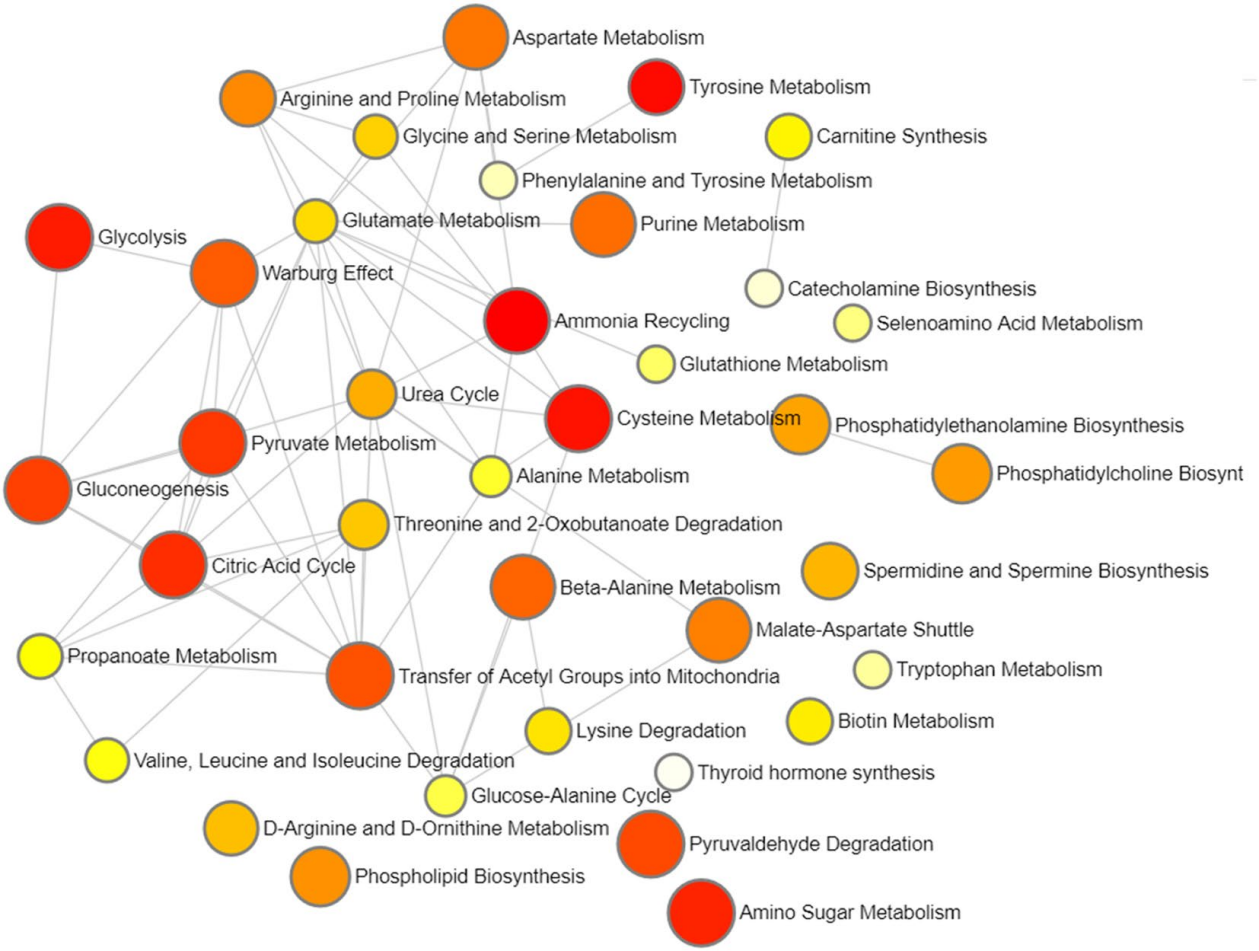
Network analysis illustrating the most significant relationships between perturbed biochemical pathways. Metabolic pathways are represented as circles according to their scores from enrichment (vertical axis) and topology analyses (pathway impact, horizontal axis). Darker circle colors indicate more significant changes of metabolites in the corresponding pathway. The size of the circle corresponds to the pathway impact score and is correlated with the centrality of the involved metabolites.

The enrichment analysis highlighted that tyrosine metabolism, pyruvate metabolism, amino sugar metabolism and cysteine metabolism, ammonia recycling, glycolysis, gluconeogenesis, and citric acid cycle were the most significantly discriminant pathways between IEI-EMF subjects and controls (Fig. 3). The network analysis demonstrated a close relationship between different metabolic pathways, for example, between glycolysis and gluconeogenesis, citric acid cycle and pyruvate metabolism, as well as between cysteine metabolism and that pyruvate metabolism via urea cycle, and tyrosine metabolism and that ammonia recycling via phenylalanine and tyrosine metabolism (Fig. 4).

## Discussion

Fibromyalgia is a complex rheumatology disease still poorly understood, characterized by non-articular diffuse musculoskeletal pain associated with allodynia and hyperalgesia^1^. In this study the plasma metabolic profile of IEI-EMF patients and healthy subjects have been compared by means of GC–MS analysis using a metabolomics approach. This population has been previously characterized for their metabolic profile by ^1^H-NMR followed by multivariate statistical analysis^8^. Results obtained showed that IEI-EMF patients presented higher plasma levels of glycine and pyroglutamate, and lower levels of 2-hydroxyisocaproate, choline, glutamine, and isoleucine compared to controls. To better define new potential reliable biomarkers to be used in the diagnostic field, in this study the profile of plasma metabolites of IEI-EMF patients using GC–MS has been defined. A total of 19 metabolites have been found differently regulated between IEI-EMF group and controls. In particular, IEI-EMF patients were characterized by higher plasma levels of 2-aminoisobutyrate, 4-hydroxyproline, alanine, glucose, leucine, lysine, proline, serine, threonine, tyrosine, urea and valine and lower levels of 2-ketohydroxicaproic acid, arabitol, aspartic acid, ethanolamine, isoleucine, ornithine, and pyruvic acid compared to controls. The observed metabolic changes can have different explanations. 2-aminoisobutyric acid (α-methylated alanine, Aib) is a non-proteinogenic alpha amino acid characterized by having the amino group attached to the carbon atom immediately adjacent to the carboxylate group (alpha carbon)^12–14^ It is widely diffused, being present in a wide range of organisms from vertebrates, including humans, to plants and insects. In 1958 Aib residues^15^ were discovered in antibiotics leading to the development of antibacterial peptides containing Aib residues as possible alternatives to antibiotics due to their broad antibacterial spectra and minimal side effects to host organisms^16,17^. A relationship between the alteration in the composition and diversity of gut microbiome and the pathophysiology of FM has been suggested, supporting the model that microbiota may affect brain activity through the gut-brain axis, with the gut representing a gateway to generalized pain^18,19^. Furthermore, alterations in microbiome composition may increase the interactions between bacteria and the intestinal immune system due to the breakdown of the intestinal barrier, favouring the release of pro-inflammatory molecules which can also increase the permeability of the blood–brain barrier^20,21^. Interestingly, the microbiome also has gut-protecting functions in the host. The fermentation of dietary carbohydrates by gut bacteria, for example, produces short-chain fatty acids (SCFAs) which are crucial to maintain the integrity of intestinal barrier^22^ and other health-related functions^23^. Recently Minerbi et al.^24^ observed a shift in the butyrate-producing bacteria species of FM patients which was associated with differences in the serum levels of SCFAs, namely butyrate and propionate. As a result, serum levels of butyric acid were higher in FM patients compared to controls, whereas levels of propionic acid and isobutyric acid were lower or showed a trend towards lower levels, respectively. On these bases, we can speculate that the increased levels of 2-aminoisobutyric acid observed in our FM patients compared to healthy subjects could be related to alterations in composition of gut microbiota. Individuals with FM are characterized by high levels of glucose^25^ and by the presence of insulin resistance (IR)^26^, which leads to sensory changes in insulin actions and insulin-mediated glucose degradation. IR can stimulate lean muscle loss, which could compromise the muscle bioenergetics in FM patients^27^. Furthermore, the high glucose level could lead to changes in the musculoskeletal contractile and electrical characteristics and could reduce the motor unit recruitment^28^ severely compromising the patient’s motility. Accordingly, our IEI-EMF patients resulted characterized by increased levels of glucose and of the branched chain aminoacids (BCAAs) leucine and valine, which are among the metabolic signature associated with IR. Indeed, recent data have shown that IR is associated to suppression of BCAA catabolism in adipose and liver tissues, via still poorly defined mechanisms, which leads to increased plasma BCAA levels, shunting BCAA catabolism to skeletal muscle^29^. Increased glucose levels in patients with fibromyalgia could also be related to increased gluconeogenesis. Therefore, as the substrates to be converted into glucose through the gluconeogenic pathway are glycerol, lactate, and pyruvic acid originating from muscle catabolism, this could justify the low pyruvic acid levels observed in IEI-EMF patients. Further supporting an increased gluconeogenesis, these patients were also characterized by high plasma levels of gluconeogenic amino acids, such as threonine, serine, and alanine. Since threonine is a potential source of serine and glycine, this could justify the increased reserve of serine and glycine evaluated in IEI-EMF patients by GC–MS or ^1^H-NMR spectroscopy^8^, respectively. Differently, Bazzicchi et al.^30^ found low plasma levels of alanine and threonine in FM patients by evaluation with a modified version of the Waters Pico Tag method. This is not surprising as a heterogeneous picture exists in the literature about amino acids levels in FM patients, possibly due to the different methods employed or the group of FM patients analyzed. The GC–MS metabolomics analysis of IEI-EMF patients also confirmed the low levels of the BCAA isoleucine and the transaminated metabolite of the BCAA leucine, 2-hydroxycaproic acid, already observed with ^1^H-NMR analysis^8^. As extensively discussed in the previous study^8^ the low plasma levels of the two metabolites could be associated with muscle weakness, manifesting abnormal fatigue and muscle tension, which represents a typical symptom of FM patients. In this study, IEI-EMF patients also resulted characterized by high plasma levels of ornithine and urea and low levels of aspartic acid which point toward an increased urea cycle activity in these subjects compared to healthy controls. The urea cycle, also known as the ornithine cycle and the Krebs-Hanseleit cycle^31^, located exclusively in the liver, has evolved in humans to remove ammonia. Based on this it could be suggested that the elevation of urea levels in IEI-EMF patients could indicate kidney sufferance. Urea and fumarate are produced while aspartate is utilized as a result of the urea cycle functioning. In line, serum levels of the non-essential amino acid aspartic acid found to be decreased in IEI-EMF patients compared to controls. Supporting an increased activity of the urea cycle, our IEI-EMF patients resulted characterized by increased levels of ornithine, which plays a central role in this cycle^31^. Besides being used as biological indicators of urea cycle functioning, aspartic acid and ornithine have many other biological roles which could affect the symptomatology of FM patients. As for aspartic acid, it is involved in several pathways: it is the precursor of several amino acids, including methionine, threonine, isoleucine, and lysine, participates in gluconeogenesis, carries reducing equivalents in the malate-aspartate shuttle, and participates in the biosynthesis of the purine and pyrimidine bases^31,32^. In addition, it plays a central role in neurological disorders. Indeed, aspartate and glutamate are considered the main excitatory amino acids in the central nervous system^32^. Thus, these amino acids have frequently been investigated in neurological disorders, such as stroke, epilepsy, and neurodegenerative disorders, and also in psychiatric syndromes, such as schizophrenia and major depression^33^. In addition, together with the inhibitory neurotransmitters glycine and gamma-aminobutyric acid, glutamate, and aspartic acid have been found to be involved in the pathogenesis of mood disorders such as the major depressive disorder (MDD)^34,35^. In particular, Lu et al.^36^ found a significant decrease in plasma aspartic acid and glutamate levels in medicine-naïve melancholic MDD patients. This was suggested to be related to the activation of hypothalamo-pituitary-adrenal (HPA)-axis and the autonomic nervous system activation, commonly associated with stress responses and depression^37,38^. The IEI-EMF patients did not show any differences from healthy control for psychological clinical characteristics. This result could be due to increased resilience and hope in subjects with chronic but non-fatal diseases. Some subjects in stress conditions develop more strategies, potentially useful to find solutions for their suffering condition^8,39^. As already mentioned, also ornithine increase may have a different explanation in FM patients, beyond that of suggesting an altered activity of the urea cycle. Ornithine, like aspartic acid, has different biological actions: plays a critical role in mitochondrial metabolic processes, is involved in the production of excess growth hormone and in burning up excess fat in the body and also plays a key role in the functioning of the liver and of the immune^40,41^. Several pathological conditions are characterized by the increase in ornithine levels over a wide range of samples^42–44^. In IEI-EMF patients high ornithine serum levels have resulted associated with increased levels of the amino acid proline and decreased levels of the proline derivative 4-hydroxyproline (Hyp) compared to healthy subjects. Together with glycine, proline and Hyp contribute to 57% of total amino acids in collagen, the most abundant protein in the body. Alterations in collagen metabolism may contribute to FM pathogenesis. One important characteristic of this chronic musculoskeletal syndrome is the tenderness at specific anatomic sites termed ‘tender points’^3^. Nerve endings located in tender points present a distinctive histological appearance involving organized collagen matrix layers, which may indicate abnormal collagen metabolism at those sites in FM patients. The remodeling of the extracellular matrix and collagen deposition around the nerve fibers, which has been suggested to contribute to the lower pain threshold at the tender points, has resulted associated with decreased levels of collagen crosslinking in FM patients^45^. This has been evidenced by lower levels of urine and serum pyridinoline:deoxypyridinoline ratios, considered as a marker of cartilage degradation, and urine Hyp, as a marker of collagen turnover, in FM patients compared to healthy controls^45^. Lower levels of Hyp and lower total concentration of the major amino acids of collagen have also been observed in the muscle tissue of FM patients compared to healthy subjects indicating a lower amount of intramuscular collagen^46^. On the other hand, an increased serum prolidase activity has been reported in FM patients, indicating a high rate of extracellular matrix remodeling which needs a high collagen turnover^47^. Indeed, prolidase catalyzes the final step in collagen degradation which completes the recycling of proline^48^ to be used in collagen biosynthesis, so that this increased activity could lead to an increased proline pool in the cells^49^. Accordingly, prolidase deficiency has been found associated with large amounts of proline excreted in the urine as iminopeptides. This has been suggested to be dependent on the block of the normal recycling of collagen due to the lack of proline liberation as a result of loss of prolidase activity^50^. On this basis, the decreased levels of Hyp and the increased proline levels observed in the serum of our IEI-EMF samples compared with healthy subjects could be ascribed to the systemic alteration in collagen metabolism associated with FM^45,47^. Also, the increased levels of lysine found in IEI-EMF patients could be related to the altered collagen turnover. Indeed, during biosynthesis collagen acquires several post-translational modifications, including lysine modifications, which are critical to the structure and biological functions of this protein. Lysine modifications of collagen are highly complicated sequential processes catalyzed by several groups of enzymes leading to the final step of biosynthesis, covalent intermolecular cross-linking^51^. Function defects in these enzymes have been found associated with several bone/skeletal disorders^52–54^, suggesting a key role for lysine modifications in bone quality. IEI-EMF patients resulted also characterized by low plasma levels of arabitol. Even if a direct association between arabitol concentration and FM is missing, several authors have shown an association between FM and dysbiosis^55^, and with small intestinal bacterial overgrowth (SIBO)^56^. Interestingly, a clinical trial with 38 FM women showed that low ingestion of fermentable oligo, di-, and monosaccharides and polyols could improve SIBO, decreasing pain associated with FM, fatigue, gastric pain, and intestinal changes^57^. On this basis, although we have no indications of the eating habits of IEI-EMF patients, we cannot exclude that their decreased arabitol plasma levels could be eventually associated with a low intake of polyols in an attempt to reduce SIBO-related symptomatology. GC–MS analysis has shown elevated plasma levels of the amino acid tyrosine in IEI-EMF patients. This result is unexpected as, similarly to alanine and threonine, a previous study by Bazzicchi et al.^30^ found reduced tyrosine plasma levels in FM patients. As tyrosine is the precursor of the catecholamines norepinephrine, epinephrine, and dopamine, its decreased levels suggested an impaired level of catecholamine synthesis in FM patients. Further supporting this hypothesis, several studies^58–60^ have reported a dopaminergic transmission dysfunction in FM patients indicating that it represents a relevant target for FM treatment. Although the reason for this discrepancy between our data and those of Bazzicchi et al.^30^ is to be elucidated, increased tyrosine plasma levels could also exert a role in FM patients. It is interesting to note that elevations of the aromatic amino acids (free tryptophan, phenylalanine, and tyrosine) and methionine have been shown to accompany chronic liver disease^61^. Notably, several currently available data have suggested that an underlying liver disease with associated psychological symptoms and sleep disturbance may function as a risk factor for FM^62^. For example, sleep disorders, psychiatric diseases, and elevated levels of inflammatory cytokines are associated with both FM and cirrhosis^63,64^. Finally, IEI-EMF patients presented decreased ethanolamine serum levels compared to healthy controls. Ethanolamine is a primary amine and primary alcohol derived from the decarboxylation of serine, and it is utilized in the synthesis of the membrane phospholipids via the CDP-ethanolamine pathway^65^. Essential for life, ethanolamine occurs in every cell in the human body as the head group of phosphatidylethanolamines (and other lipids) and it is present as free ethanolamine at varying concentrations in body fluids^66^. Mammals cannot synthesize ethanolamine, but it is obtained from the diet as free ethanolamine or in the form of phosphatidylethanolamine, which is degraded by phosphodiesterases to generate glycerol and ethanolamine^67^. Other sources of ethanolamine or phosphoethanolamine in the human body are the degradation of sphingosine phosphate by sphingosine phosphate lyase and the degradation of the endocannabinoid anandamide (AEA), also known as arachidonoylethanolamide, by the fatty acid amine hydrolase (FAAH)^68^. Interestingly, FM patients have been found characterized by increased plasma levels of AEA compared to healthy subjects^69^. The endocannabinoid AEA is a lipid mediator of the endocannabinoid system which is well characterized as an activator of the cannabinoid receptors (CBR1 and CBR2). The endocannabinoid system is associated with multiple biochemical actions by modulating, for example, pain, inflammation, and emotions, anxiety, and stress^70,71^. The high plasma levels of AEA observed in FM patients^69^ have been suggested to represent a non-specific phenomenon reflecting a compensatory adaptation to stress pain, as demonstrated by its occurrence in several pathological conditions^72^**,**. Though the pathophysiological relevance of the high AEA levels in FM is still unclear, animal models have suggested a role for endocannabinoids in buffering stress reaction through stress-induced analgesia mediated via central and peripheral CB1R receptors. Furthermore, since AEA is a known central neuromodulator involved in the extinction of traumatic memories^73^, it has been speculated that the elevation of its plasma levels could contribute to the change in brain structures that characterize FM patients^74,75^. It could be hypothesized that increased AEA levels in FM patients could originate as an autoprotective mechanism to cope with chronic ongoing stress, but, when AEA production is sustained due to repetitive stress, AEA-mediated CB1R down-regulation could lead to exacerbation of the stress-related symptomatology, as suggested for persons with repetitive childhood trauma^76^. On this basis, as ethanolamine is a precursor of AEA synthesis, its low levels in IEI-EMF patients could be explained by its consumption to form an increased content of AEA molecules compared to healthy subjects. Alternatively, as ethanolamine could be produced by FAAH-mediated AEA degradation^67^, its decreased serum concentration in FM patients could reflect a decreased enzyme expression/activity which could result in increased serum AEA content. According to this, in subjects characterized by FAAH C385A polymorphism, a missense functional variant with a significant reduction in enzymatic activity, a higher AEA concentration was observed compared to controls^77^.

## Conclusion

Our previous study showed a different plasma metabolic profile between IEI-EMF patients and healthy subjects analyzed by ^1^H-NMR spectroscopy coupled with multivariate statistical analysis^8^. The metabolites identified as differentially expressed between the two groups were mainly related to oxidative stress defense, pain mechanisms, and muscle metabolism. The current study using GC–MS analysis in plasma samples from the same population profiled intra-individual changes in the levels of 19 metabolites. The presence of high levels of circulating indicators of IR (glucose, leucine, and valine) and gluconeogenesis (threonine, serine, and alanine) could justify the low pyruvic acid and the high glucose levels found in FM patients which could affect the musculoskeletal system efficiency. Supporting the paradigm that regulation of bone and muscle activity is affected in FM patients, the present results are characterized by altered levels of amino acids related to collagen turnover (proline, Hyp, and lysine) and muscle performance (isoleucine and 2-ketohydroxycaproic acid). Altogether the results suggest a possible metabolic alteration in FM associated with electrosensitivity.

## Materials and methods

### Characteristics of the study population

The study was carried out on 54 subjects: 31 affected by FM and electromagnetic sensitivity (IEI-EMF) (30 females and 1 male), and 23 controls (21 females and 2 males). The demographic characteristics of the population under study and their dietary habits were evaluated to identify intolerance (or food avoidance) have been previously reported^8^. The patients included in the study were previously diagnosed with FM. The presence of FM was ascertained by a questionnaire according to the American College of Rheumatology (ACR) criteria^3^, as already reported8. Individuals with IEI-EMF were identified according to the inclusion criteria reported in the study of Baliatsas et al.^9^. Personal characteristics and electromagnetic/chemical exposure of the subjects at home or work were used to classify the triggering events, and the exclusion criteria for both patients and control subjects and also reported in the study of Piras et al. ^8^. Furthermore, each subject was asked to compile the second, third, fourth and fifth scale of PAI^78^ to assess clinical and psychological characteristics such as anxiety, depression, anxiety correlated disorders (e.g., OCD, phobias, etc.) and emotional instability. This test is useful to preliminary assess psychological pathologies in the patients. The study has been conducted according to the Declaration of Helsinki and has been approved by the institutional ethics committee of the University of Cagliari, Italy. Written informed consent was obtained from all the study participants.

### GC–MS sample preparation and analysis

The plasma was obtained from whole blood samples collected in tubes with EDTA from fasting IEI-EMF patients and control subjects and subsequently centrifuged at 2000 rpm for 10 min. Then, the plasma was stored at -80 °C until use. 400 µL of plasma were extracted with a modified Folch method. Briefly, 600µL of methanol, 600µL of chloroform and 175µL of Milli-Q water were added to 400µL of each plasma sample.. After centrifugation at 4500 rpm for 20 min at 4 °C, the hydrophilic and lipophilic phases were separated. 200 µL of the water-phase for each sample was concentrated overnight in a speed-vacuum. Blanks were made following the same procedure used for the samples to avoid noises due to the chemicals used for the preparation and the laboratory instruments. Derivatization was made by adding to dried samples 100μL of methoxamine hydrochloride in pyridine solution (10 mg/mL) for 17 h. Subsequently, 100μL of N-trimethylsilyltrifuoroacetamide (MSTFA) were added and vortexed at R.T., 1 h. Samples were then diluted in hexane (600μL) with an internal standard (undecane at 25 ppm). Diluted samples were then filtered (PTFE 0.45 μm) and transferred into glass vials. A 1 μL aliquot of the samples was injected splitless by an autosampler in an Agilent 7890A gas chromatograph coupled with an Agilent 5975 C mass spectrometer equipped with a HP-5MS capillary column (5%-Phenyl-methylpolysiloxane; 30 m, 25 mm i.d., 0.25 μm film thickness). The initial oven temperature was 50 °C (hold 3 min) and increased at 10 °C/min to 250 °C for a total run of 35 min. Spectra were acquired in electron impact mode and full scan monitoring mode (m/z 50–800). The injector and ion source temperature were respectively set at 200 and 250 °C. Helium was used as the carrier gas in constant pressure mode (7.6522psi). The identification of metabolites was performed using the standard NIST 08 and Golm Metabolome Database (GMD) mass spectra libraries, as well as by comparison with authentic standards. A representative GC–MS chromatograms obtained from controls and IEI-EMF patients was reported in Figure S5. The R library XCMS was used for peak detection and retention time correction. Parameters utilized for peak deconvolution for GC–MS matrices were manually optimized^79^.

### Multivariate statistical analysis

A multivariate statistical analysis was performed using SIMCA software (Version 16.0, Sartorius Stedim Biotech, Umea, Sweden). Raw data were organized in matrices, and datasets were normalized using Median Fold Change and scaled with unit variance (UV) scaling. A PCA was performed in the data set to evaluate the homogeneity of the samples and identify any possible trends and/or outliers in the dataset^80^. Subsequently an OPLS-DA analysis was conducted to reduce model complexity and to better highlight sample discrimination. The variance and predictive ability (R2X, R2Y, and Q2) were established to evaluate the suitability of the models. A permutation test (n = 500) was performed to validate the models^8^. Metabolites with a Variables Important in the Projection (VIP) value higher than > 1 were selected for evaluation of their role in class separation.

### Univariate statistical analysis for GC–MS data

GraphPad Prism software (version 7.01, GraphPad Software, Inc., CA, USA) was used to perform the univariate statistical analysis of the data. The statistical significance of the differences in metabolite concentrations was calculated by using the Mann–Whitney U test and a *p*-value < 0.05 was considered statistically significant. To acquire the level of significance for multiple testing, the Benjamini–Hochberg adjustment was applied to the obtained *p*-values^81^. To further evaluate the diagnostic robustness of potential biomarkers, receiver operating characteristic (ROC) was carried out. The Linear SVM algorithm was used to construct the ROC. MetaboAnalyst 5.0 *(*https://www.metaboanalyst.ca*)* program was used to perform the Enrichments and Network analysis, respectively.

## Supplementary Information

Supplementary Information.

## Data Availability

The datasets used and/or analyzed during the current study available from the corresponding author on reasonable request.
