# Supplementary material for: Metabolomics analysis of plasma samples of patients with fibromyalgia and electromagnetic sensitivity using GC–MS technique
Source: Sci Rep. 2022 Dec 19;12:21923. doi: 10.1038/s41598-022-25588-2 (PMC9763344; doi:10.1038/s41598-022-25588-2)
Supplement: Supplementary file 1 — Supplementary Information. [file 41598_2022_25588_MOESM1_ESM.docx]

**Metabolomics analysis of plasma samples of patients with fibromyalgia and electromagnetic sensitivity using GC-MS technique**

Cristina Piras^1^, Monica Pibiri^1^, Stella Conte^2^, Gabriella Ferranti^2^, Vera Piera Leoni^1^, Sonia Liggi^3^, Martina Spada^1^, Sandro Muntoni^1^, Pierluigi Caboni^4^, Luigi Atzori^1^**^*^**

^1^ Department of Biomedical Sciences, University of Cagliari, Cagliari, Italy

^2^ Department of Education, Psychology and Philosophy, University of Cagliari, Cagliari, Italy

^3^ Department of Metabolism, Digestion and Reproduction, Imperial College London, London, UK

^4^ Department of Life and Environmental Sciences, University of Cagliari, Cagliari, Italy

***Corresponding Author:** Prof. Luigi Atzori, MD, PhD; Department of Biomedical Sciences; Clinical Metabolomics Unit; University of Cagliari; Blocco A, Cittadella Universitaria, Monserrato (CA); Italy; Phone: +39 0706758390; email: latzori@unica.it

**Keywords:** Metabolomics; GC-MS; Fibromyalgia; Electromagnetic sensitivity; Biomarkers.

**TABLE.**

**Table S1**. PAI test (Mean and Standard Deviation*)

| **a)** | **IEI-EMF*** | | **Controls*** | |
| --- | --- | --- | --- | --- |
| Anxiety | 48.48 (±18.69) | | 50.62 (±19.55) | |
| Related anxiety disorders | 26.64 (±8.58) | | 27.95 (±9.27) | |
| Depression | 62.87 (±25.99) | | 62.54 (±31.33) | |
| Mania | 59.00 (±17.54) | | 57.00 (±22.29) | |
|  |  | |  | |
| **b)** | **F**** | **DF**** | | **P**** |
| Lambda by Wilks | 0.940 | 4/50 | | 0.528 |
| Anxiety | 0.367 | 1/53 | | 0.542 |
| Related anxiety disorders | 0.995 | 1/53 | | 0.323 |
| Depression | 0.082 | 1/53 | | 0.775 |
| Mania | 0.03 | 1/53 | | 0.959 |

****F:** F-test ; **DF:** degree freedom; **P:** p-value

**Table S2.** Relative concentrations of identified metabolites in IEI-EMF patients compared with controls. Data are expressed as means and standard error

| ***Metabolites*** | **Controls**  (Mean ±SE) | **IEI-EMF**  (Mean ±SE) |
| --- | --- | --- |
| Lactic acid | 1295647±656656 | 1103601±158905 |
| **Pyruvic acid** | **74613±18905** | **24059±2200** |
| Glycolic acid | 17775±2135 | 16080±1922 |
| **Alanine** | **2698722±374251** | **3717569±269005** |
| α-Hydroxybutyric acid | 304085±30145 | 302058±23376 |
| m-Cresol | 129163±21150 | 180033±20010 |
| β-Hydroxybutyric acid | 1223380±155961 | 1149137±241052 |
| **2-Aminoisobutyrate** | **71898±11429** | **130355±10216** |
| **Proline** | **1213641±181101** | **1680966±136022** |
| **Valine** | **1192086±138583** | **1797340±106123** |
| **2-Ketohydroxycaproic** | **40362±5165** | **24978±2667** |
| **Urea** | **6113247±1203627** | **9283302±894049** |
| **Ethanolamine** | **2009136±176575** | **1406502±86173** |
| **Serine** | **327969±54815** | **480255±37843** |
| **Leucine** | **743382±92209** | **1067711±68861** |
| Glycerol | 2054535±164147 | 2092400±114231 |
| **Isoleucine** | **382979±37605** | **282968±23169** |
| **Threonine** | **430508±61208** | **715748±64739** |
| Glycine | 2327729±333230 | 31002129±332200 |
| Succinic acid | 323653±43218 | 261004±28350 |
| Glyceric acid | 300783±27892 | 255784±19253 |
| **Aspartic acid** | **159911±29904** | **652223±8192** |
| [**4-Hydroxyproline**](https://hmdb.ca/metabolites/HMDB0000725) | **32329±2543** | **58286±6038** |
| Malic acid | 66007±13927 | 67289±7184 |
| Threitol | 333140±89099 | 397605±312731 |
| Pyroglutamic acid | 422060±97138 | 670536±93245 |
| Threonic acid | 125647±7846 | 111553±12412 |
| Creatinine | 85030±15002 | 72552±9795 |
| Phenylalanine | 273614±17652 | 275614±1148 |
| **Ornithine** | 141166±268265 | 53690±8041 |
| Glutamine | 85530±12548 | 89895±11359 |
| Lauric acid | 202636±19208 | 483186±178453 |
| **Arabitol** | **120763±11507** | **79713±4455** |
| Citric acid | 412806±57124 | 501266±43887 |
| 1,5-Anhydroglucitol | 2105164±274239 | 2126985±146994 |
| Fructose | 4698619±450281 | 6655654±724476 |
| Mannose | 997341±85655 | 1784416±748327 |
| **Glucose** | **577372±52534** | **770568±57586** |
| **Tyrosine** | **576400±48529** | **766301±59884** |
| **Lysine** | **28835±4583** | **50638±7113** |
| Inositol | 32172±8423 | 28047±6006 |
| Maltotriose | 362387±32693 | 398780±25745 |
| Palmitic acid | 5367828±496119 | 5342382±589693 |
| Myo-inositol | 897128±54512 | 883419±47212 |
| Uric acid | 86266±17637 | 56050±12957 |
| Tryptophan | 76817±14449 | 92124±15039 |

**FIGURES.**

**
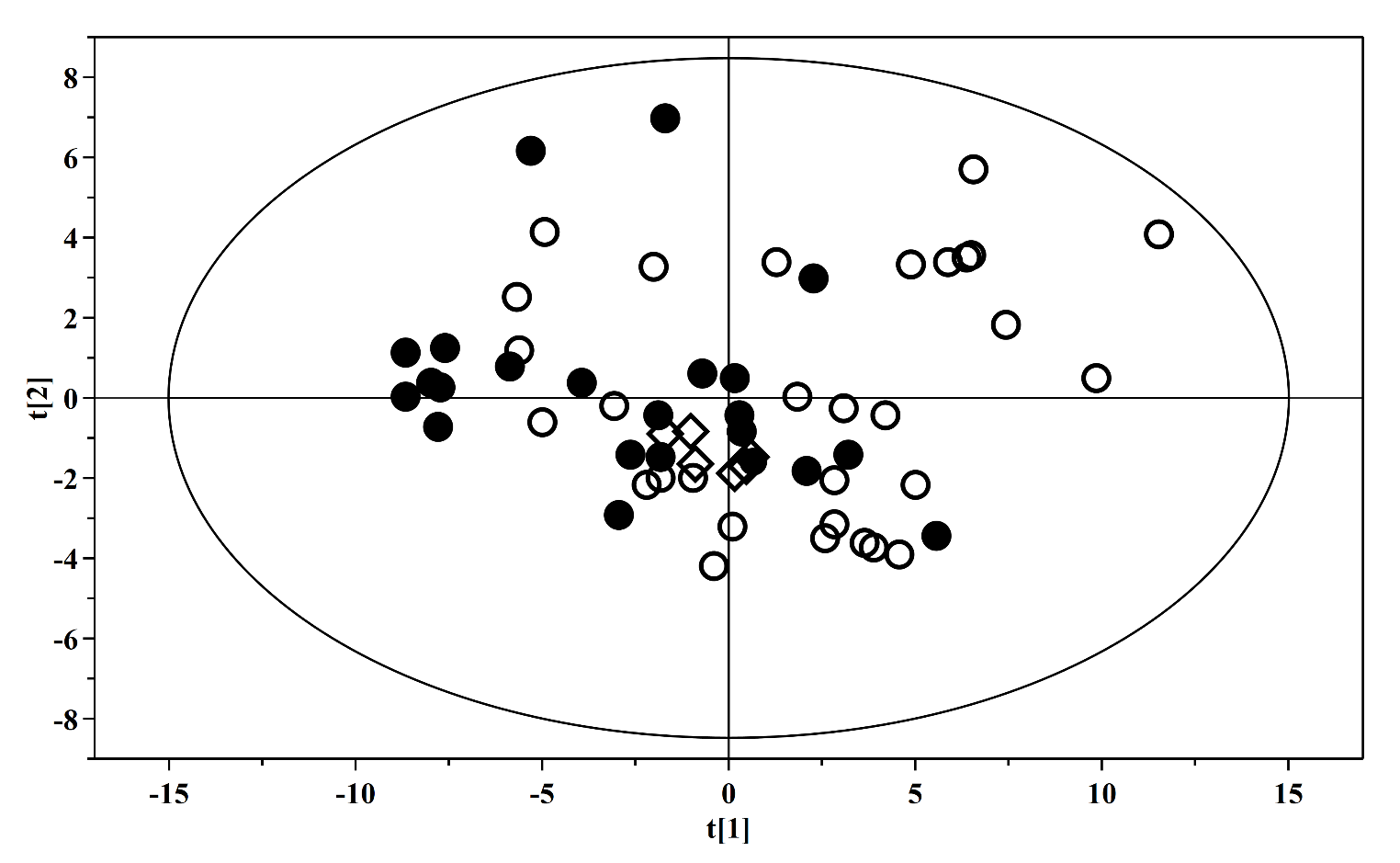
**

**Figure S1.** PCA scores plot: controls (full circle), IEI-EMF subject (open circle) and QC (diamond). The PCA was built with two PCs (PC1= 0.386; PC2= 0.121).

**
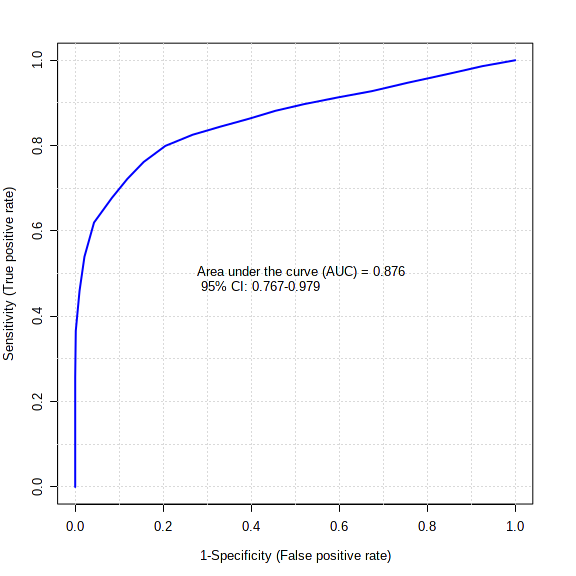
**

**Figure S2.** Receiver Operating Characteristic (ROC) plot built by combining all significantly altered metabolites between IEI-EMF subjects and controls. The figure was drawn via MetaboAnalyst software v 5.0 *(https://www.metaboanalyst.ca/)*.


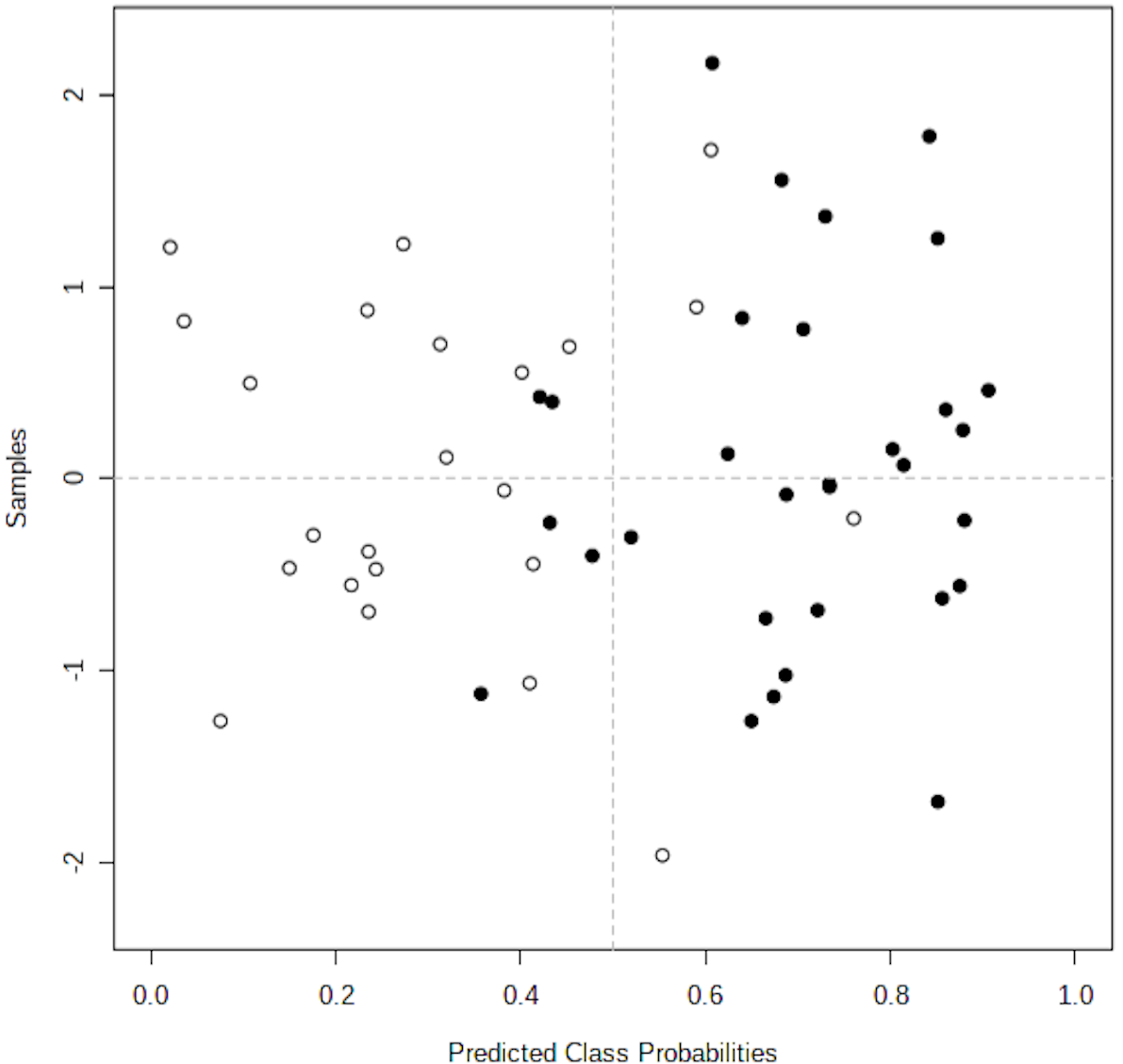


**Figure S3.** The predicted class probabilities (average of the cross-validation) for each sample using the 19 features model of metabolites contributed to the prediction model ranked by mean importance measure. Controls (open circle); IEI-EMF subjects (full circle).


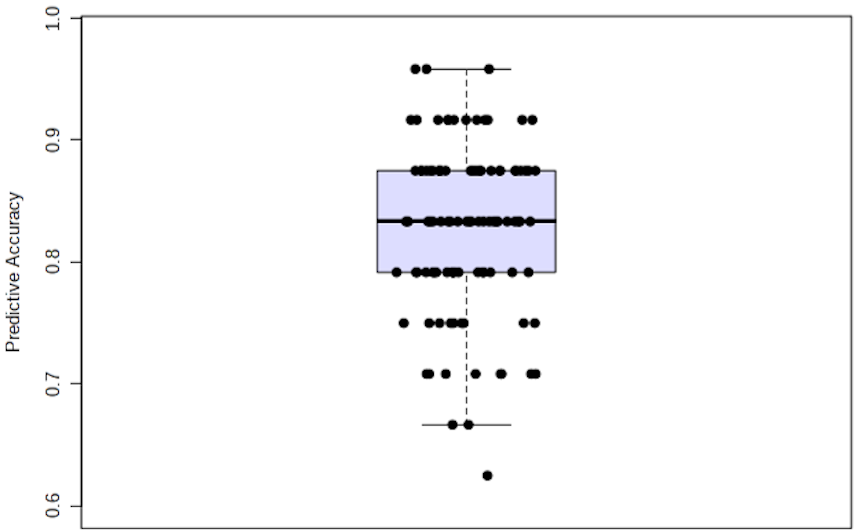


**Figure S4.** Box plot of the predictive accuracy (with an average of 0.827) of the biomarker model based on 100 cross validations.


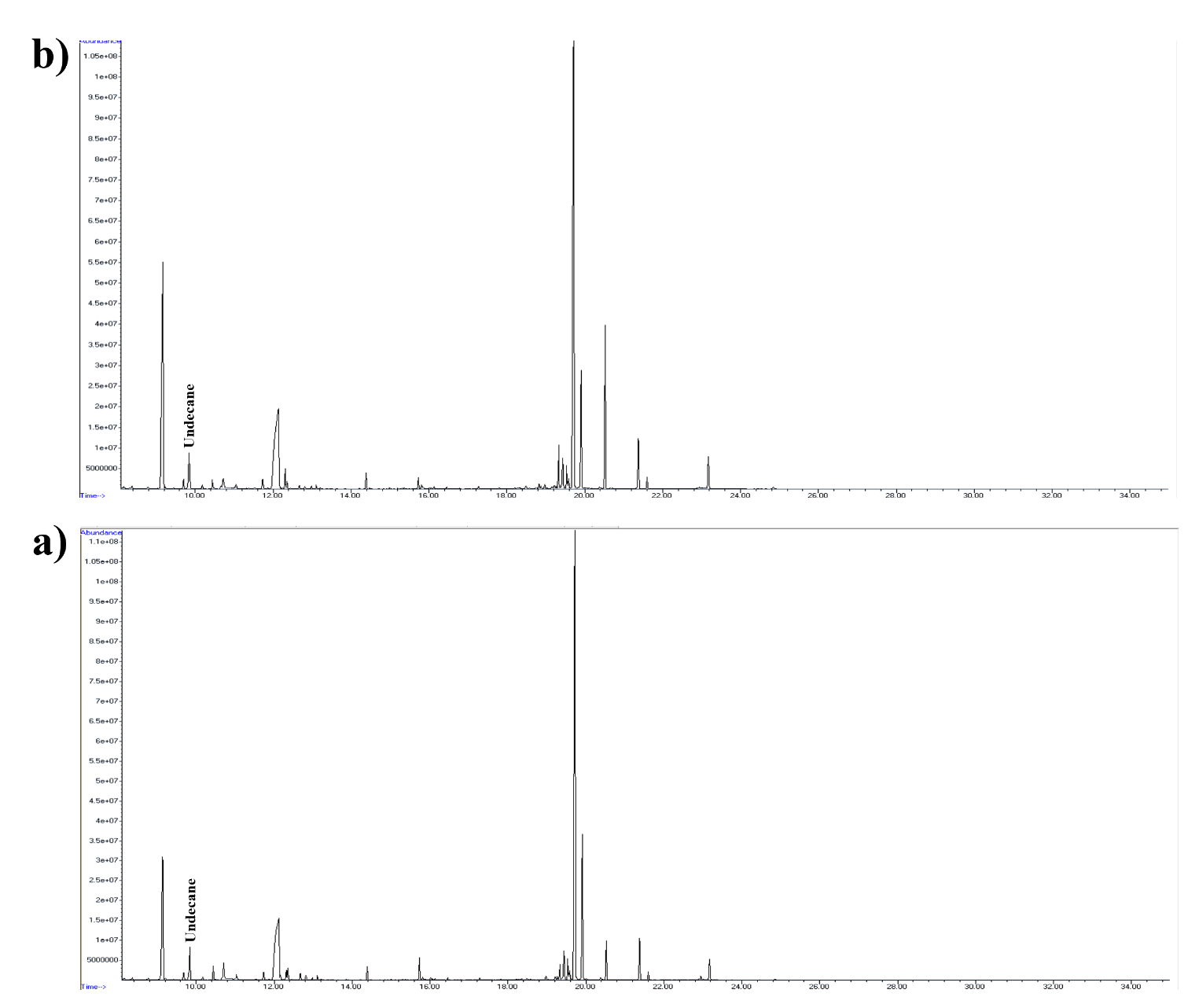


**Figure S5**. GC-MS chromatograms obtained from a) control and b) IEI-EMF patients.
